# Multiple direct and indirect mechanisms drive estrogen-induced tumor growth in high grade serous ovarian cancers

**DOI:** 10.18632/oncotarget.6943

**Published:** 2016-01-18

**Authors:** Alessandra Ciucci, Gian Franco Zannoni, Marianna Buttarelli, Lucia Lisi, Daniele Travaglia, Enrica Martinelli, Giovanni Scambia, Daniela Gallo

**Affiliations:** ^1^ Department of Obstetrics and Gynecology, Catholic University of The Sacred Heart, 00168 Rome, Italy; ^2^ Department of Histopathology, Catholic University of The Sacred Heart, 00168 Rome, Italy; ^3^ Institute of Pharmacology, Catholic University of The Sacred Heart, 00168 Rome, Italy

**Keywords:** ER, estradiol, ovary, tumor-associated macrophages, tumor microenvironment

## Abstract

The notion that menopausal estrogen replacement therapy increases ovarian cancer risk, but only for the two more common types (i.e. serous and endometrioid), while possibly decreasing risk for clear cell tumors, is strongly suggestive of causality. However, whether estradiol (E_2_) is tumorigenic or promotes development of occult preexisting disease is unknown. The present study investigated molecular and cellular mechanisms by which E_2_ modulates the growth of high grade serous ovarian cancer (HGSOC). Results showed that ERα expression was necessary and sufficient to induce the growth of HGSOC cells in *in vitro* models. Conversely, *in vivo* experimental studies demonstrated that increasing the levels of circulating estrogens resulted in a significant growth acceleration of ERα-negative HGSOC xenografts, as well. Tumors from E_2_-treated mice had significantly higher proliferation rate, angiogenesis, and density of tumor-associated macrophage (TAM) compared to ovariectomized females. Accordingly, immunohistochemical analysis of ERα-negative tissue specimens from HGSOC patients showed a significantly greater TAM infiltration in premenopausal compared to postmenopausal women. This study describes novel insights into the impact of E_2_ on tumor microenvironment, independently of its direct effect on tumor cell growth, thus supporting the idea that multiple direct and indirect mechanisms drive estrogen-induced tumor growth in HGSOC.

## INTRODUCTION

Ovarian cancer is the most deadly gynecologic malignancy, with more than 140,000 women dying from this cancer across the world in 2008 [1]. Over 90% of ovarian malignancies are categorized as epithelial ovarian cancers, and currently five main types are identified: high-grade serous carcinoma (70%), low-grade serous carcinoma (<5%), mucinous carcinoma (3%), endometrioid carcinoma (10%), and clear-cell carcinoma (10%). These types are essentially distinct diseases, as indicated by differences in epidemiological and genetic risk factors, precursor lesions, patterns of spread, molecular events during oncogenesis, response to chemotherapy, and prognosis [2]. The most common and lethal of all ovarian cancer subtypes are the high grade serous carcinomas (HGSOCs), arising within the ovarian surface epithelium (OSE) or the fallopian tube surface epithelium [3]. Debulking surgery and platinum-based chemotherapy is the standard treatment for HGSOCs and 75% of the women may not have any evidence of disease at the end of this treatment. However, most tumors will relapse within 18 to 28 months and only 20% to 40% of all women will survive beyond five years [4]. Innovative and more effective treatment strategies are thus required to improve quality and duration of life for women with this disease.

In this context, there are preclinical and clinical evidences increasingly supporting a role of estrogen-regulated pathways in the etiology and progression of ovarian cancer [5], although comprehensive mechanistic studies are lacking. Noteworthy, estrogen signaling appears to operate differently in the distinct ovarian malignancy subgroups, due to their intrinsic heterogeneity. Indeed, a very recent meta-analysis of 52 epidemiological studies demonstrated an increased ovarian cancer risk in hormone-therapy users, but only for the two more common types (i.e. serous and endometrioid), while risk was possibly decreased for clear cell tumors [6]. On the other hand, in premenopausal years, the use of oral contraceptives has been reported to decrease serous, endometrioid and clear cell, but not mucinous tumors [7]. The reasons for these findings are unclear, but these data are overall strongly suggestive of causality.

It is known that biological actions of estrogens are largely mediated by two distinct estrogen receptor isoforms, namely ERα and ERβ, members of the nuclear steroid receptor (NR) superfamily [8]. More recent studies have revealed that estrogens also mediate rapid signaling events traditionally associated with G protein-coupled receptors, and the G protein-coupled estrogen receptor GPER (formerly GPR30) has now become recognized as a mediator of estrogen's rapid cellular effects throughout the body [9]. In the normal ovary, the levels of ERβ are high and predominate over ERα, being ERβ1, -β2, and -β5 the most represented isoforms [10, 11], whereas an opposite pattern characterizes the development of ovarian cancer [8]. Besides, ERβ and its isoforms may have different roles and be associated with distinct prognosis depending on their cellular localization. Indeed, we recently showed that in advanced HGSOC, high cytoplasmic ERβ2 expression may define patients with aggressive biology and possibly resistant to chemotherapy, providing also *in vivo* mechanistic evidence for an anti-apoptotic function of mitochondrial ERβ2 [12, 13]. Finally, normal ovaries also express GPER [14] while in ovarian cancer protein overexpression predicts poor survival [15, 16].

Preclinical studies have indicated a promoting effect of estrogens on ovarian cancer growth in cell cultures and *in vivo* models, this effect being reported as mainly mediated by ERα and GPER. Ligand-activated ERα induces expression of genes involved in cell survival and proliferation, while GPER-mediated proliferative effects mostly involve the activation of growth factor receptor transduction pathway; conversely, the function of ERβ1 has been found to be anti-proliferative and pro-apoptotic [5, 8, 9, 17].

Despite all these clinical and experimental data showing the importance of estrogens in the development and progression of ovarian cancer, many questions still remain and, among these, the contribution of 17β-estradiol signaling through ERα, ERβ isoforms and GPER is likely to be really complex and specific to particular cell types, tissues, ligands and diseases, thus deserving additional studies. The present study aimed at evaluating the impact of 17β-estradiol on HGSOC, providing novel insights into its functional role in promoting the growth of ERα-negative and ERα-positive cancers by induction of dynamic changes in the composition and function of the surrounding and supportive stroma.

## RESULTS

### Expression of steroid hormone receptors in a panel of HGSOC cell lines

**Figure 1:**
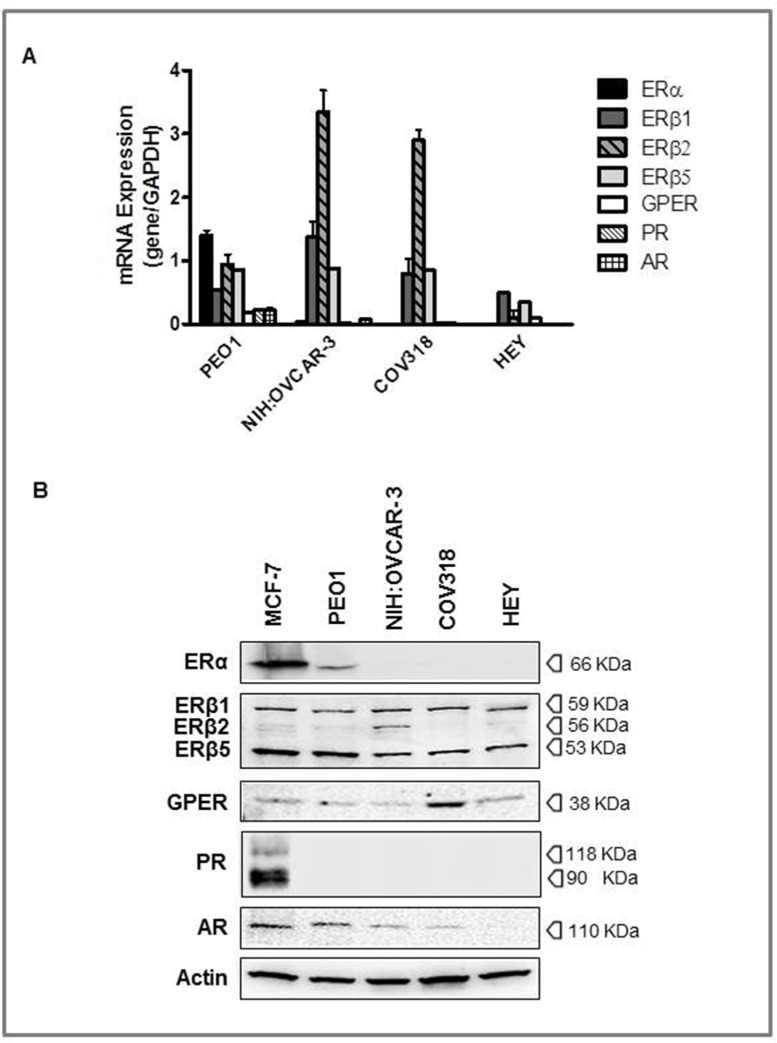
Expression of steroid hormone receptors in high grade serous ovarian cancers (HGSOC) cell lines PEO1, NIH:OVCAR-3, COV318, and HEY cells were grown in complete culture medium and MCF-7 cells were used as positive control. **A.** The relative mRNA expression of hormone receptors was evaluated by RT-PCR, utilizing a specific sets of primers (see Table I). All samples were normalized to the housekeeping gene, GAPDH. The results are presented as fold change for each mRNA in HGSOC cell lines compared to MCF-7 cells. **B.** Representative Western blot of hormone receptors expressions. Protein levels were determined by subjecting 60 μg of protein extract to SDS-gel electrophoresis, followed by Western blotting using specific antibodies. β-actin was used as a control of equal sample loading.

To the aim of the study, we selected 4 different ovarian cancer cell lines among those indicated as really representative of HGSOC lesions [18, 19] and evaluated the expression of hormone receptors by RT-PCR and WB analyses (Figure 1). MCF-7 cells were used as positive control.

Results obtained showed ERα mRNA expression in PEO1, and, at a very low levels, in NIH:OVCAR-3, whereas COV318 and HEY cells were negative. On the other hand, all cell lines were shown to express the three ERβ transcript variants tested (i.e. ERβ1, ERβ2 and ERβ5), and GPER as well (low levels). Progesterone receptor mRNA was detected only in PEO1 and, to a lesser extent, in COV318, while AR transcripts were shown in PEO1 and NIH:OVCAR-3 cells only. There was a concordance between gene and protein expression for GPER and for the three ERβ isoforms, these latter identified with the ERβ H150 antibody, as previously reported [20]. Conversely, ERα protein was detected only in PEO1, and PR was undetectable in all ovarian cancer cell lines (Figure 1). Finally, the androgen receptor protein was found in all but HEY cells. In keeping with previous literature [21–24], we found a lack of ERα and PR expression in NIH:OVCAR-3, although other authors described this cell line as steroid hormone-receptor positive [25–27], this raising the possibility that different clones may have been selected over time in different laboratories.

Overall these findings while reinforcing the concept that mRNA levels cannot be used as surrogates for corresponding protein levels without verification, mostly important show that the hormone receptors status varies among the different cell lines, thus reflecting the considerable heterogeneity observed in clinical HGSOC specimens [12, 28].

### Regulation of HGSOC cell growth by selective ligands

To enable us to relate ER status to estrogen responsiveness, proliferation of HGSOC cells was evaluated 120 hours following treatment with E_2_, selective ERs/GPER agonists (i.e. PPT, DPN or G1) or, when relevant, with the ER antagonist ICI 182,780. MCF-7 cells were used as positive control.

**Figure 2:**
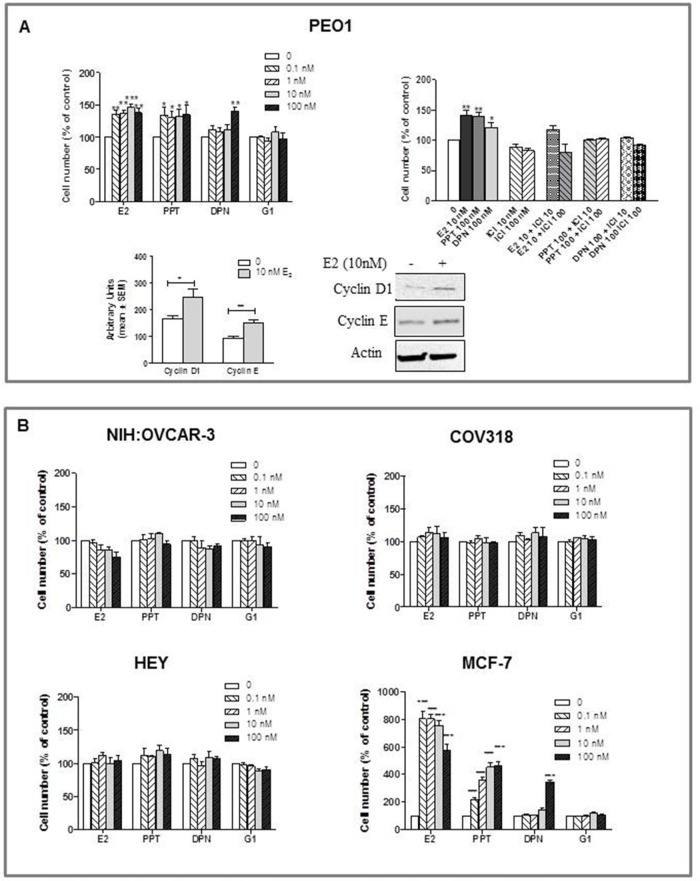
Effects of E_2_, the ERα-selective agonist PPT, the ERβ-selective agonist DPN, and the GPER-selective agonist G1 on the growth of HGSOC cell lines (PEO1, HEY, COV318 and NIH:OVCAR-3) Cells were treated with various concentrations of substances in phenol-red free medium supplemented with charcoal stripped FBS. Concentrations are expressed in nanomolar. Control cells received the same amount of diluent. The medium was renewed after 48 hours. At 120 hours of incubation viable cells were counted using Nucleocounter. All results are expressed as the mean ± SEM derived from at least three different experiments. **A.** In PEO1 cells growth was modulated by E_2_ or selective agonist treatment, an effect reverted by the ER antagonist ICI 182,780 (10 and 100 nM) (*P<0.05, **P<0.01, ***P<0.001). To confirm the E_2_-induced modulation of cell proliferation, cyclin D1 and cyclin E was evaluated by western blot analysis after 120 hours treatment. Quantitated protein levels were normalized to β-actin (*P<0.05, **P<0.01). **B.** The proliferation of NIH:OVCAR-3, COV318 and HEY was not modulated by either the endogenous or the selective synthetic ligands. MCF-7 cells were used as positive control.

None of the cell lines examined showed to be estrogen-dependent for growth *in vitro*. However, while PEO1 demonstrated estrogen-sensitivity (Figure 2A), proliferation of NIH:OVCAR-3, COV318 and HEY was not modulated by either the endogenous or the selective synthetic ligands (Figure 2B). Specifically, Figure 2A shows that the ERα-positive PEO1 cell line was significantly growth-stimulated by E_2_ and PPT relative to a control sample, cell proliferation being accompanied by an increase in cyclin D1 and E levels, as shown by Western blot analysis (Figure 2A). As expected, this E_2_-induced stimulation of cell growth was competitively blocked by ICI 182,780, which was also able to reverse the growth stimulatory effect induced by the selective ERα-agonist, PPT. The increased growth observed at 100 nM DPN was considered to be compatible with the ERα-transactivation when high dose of this ERβ-selective agonists are used [29], and was again reversed by ICI 182,780 (Figure 2A). MCF-7 cells used as positive control gave results consistent with literature data (Figure 2B) [30, 31].

Overall, these results indicate that ERα expression is necessary and sufficient to induce the E_2_-stimulated growth of HGSOC cells in *in vitro* models, while E_2_ treatment of ERα-negative cell lines determine a lack of effect on cell proliferation.

### *In vivo*-Effect of E_2_ treatment on ovarian cancer growth

*In vivo* experiments using the NIH:OVCAR-3 and the HEY ovarian cancer models were carried out to assess the role of estrogens on *in vivo* ovarian cancer growth. Female mice were ovariectomized at 6 weeks of age and divided into two different groups, i.e. Ovx and Ovx+E_2_. Treatment with 17β-estradiol started one week after ovariectomy and three days after, xenografts were established by injecting either NIH:OVCAR-3 (s.c. implantation) or HEY (i.p. implantation) cells into both Ovx and E_2_-treated females.

### NIH:OVCAR-3 study

**Figure 3:**
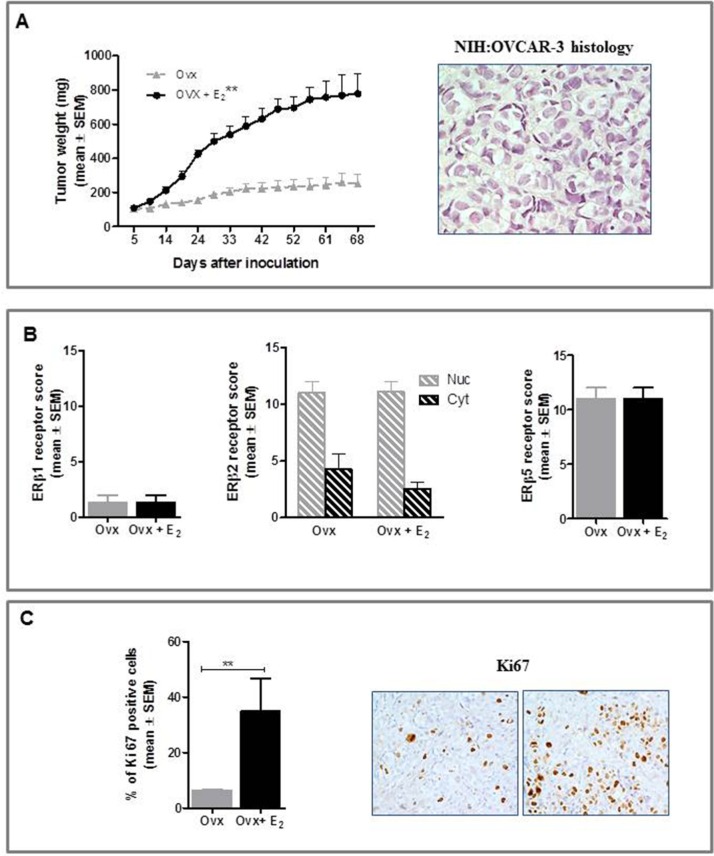
Effect of E_2_ on in vivo growth of NIH:OVCAR-3 in female BALB/c nude mice **A.** E_2_ stimulated the s.c. growth of NIH:OVCAR-3 compared to Ovx females (**P<0.01, n=8 mice/group). Histological features of the tumor (magnification 40x). **B.** Immunohistochemical analysis did not show any treatment-related difference in expression of the three ERβ isoforms (n=8 tumors/group). Nuc, nuclear and Cyt, cytoplasmic expression. **C.** Tumors in E_2_-treated females were characterized by a higher proliferative index (**P<0.01, n=8 tumors/group). Representative images for Ki67 immunostaining of tumors from Ovx and Ovx+E_2_ mice (magnification 40x).

Ovx females had significantly lower relative uterus weight (0.7±0.04 mg/g body weight) than E_2_-treated group (6.1±0.2 mg/g body weight, p<0.001), this confirming that ovariectomy and E_2_ replacement were successful. As shown in Figure 3A, E_2_-treated mice showed significantly increased tumor growth rates than Ovx (P<0.01), histology of NIH:OVCAR-3 xenografts being consistent with that of poorly differentiated HGSOC. To shed light on the mechanisms for a potential influence of estrogens on *in vivo* growth of HGSOC cells we examined by immunohistochemical analysis hormone receptor expression profile in tumors from both groups. In line with WB results on cellular extracts, we found no detectable ERα and PR in epithelial malignant cells from both groups (data not shown), whereas revealing ERα staining in stromal cells. Expression of ERβ1 and ERβ5 was mainly nuclear, while ERβ2 staining was also observed in the cytoplasmic compartment, with no differences between Ovx and Ovx+E_2_ mice (Figure 3B). Likewise, GPER protein levels did not change following E_2_ treatment (data not shown). Finally, immunohistochemical assessment of proliferation rate showed that Ki67 positive cells were significantly higher in Ovx+E_2_ females (35 ± 11%) than in Ovx controls (6.5 ± 0.3%) (P<0.01, mean ± SEM, Figure 3C).

### HEY study

**Figure 4:**
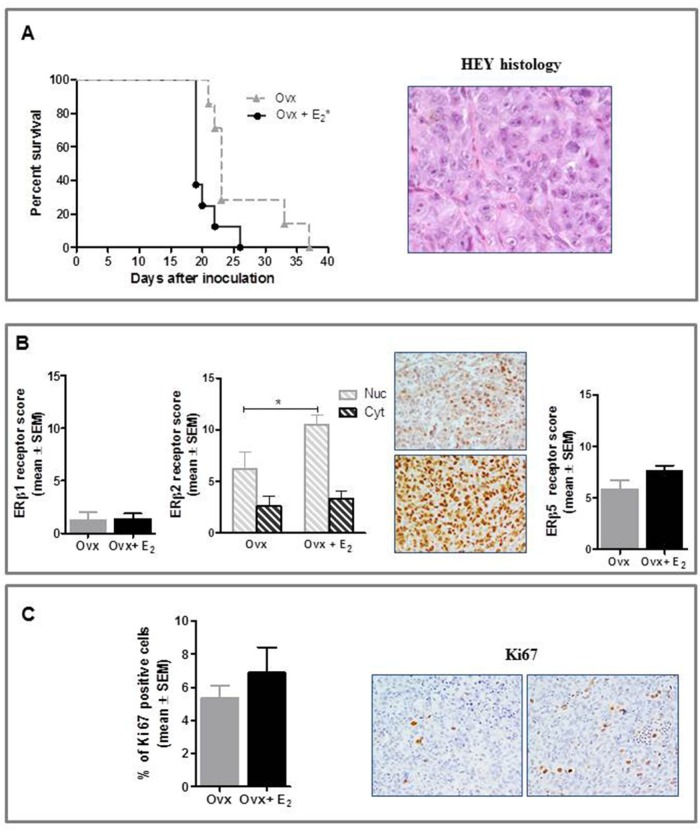
Effect of E_2_ on in vivo growth of HEY cells in female BALB/c nude mice **A.** E2 stimulated the i.p. growth of HEY compared to Ovx females (*P<0.05, n=8 mice/group). Histological features of the tumor (magnification 40x). **B.** Immunohistochemical analysis did not show any treatment-related difference in ERβ1 and ERβ5 expression, while nuclear ERβ2 was increased in E_2_-treated mice (*P<0.05, n=8 tumors/group). Representative images for ERβ2 immunostaining from Ovx and Ovx+E_2_ tumors (magnification 40x). Nuc, nuclear and Cyt, cytoplasmic expression. **C.** Immunostaining for Ki67 was higher in tumors of E_2_-treated than Ovx females, although this difference was not statistically significant (n=8 tumors/group); representative stained section of tumors from Ovx and Ovx+E_2_ mice are shown (magnification 40x).

Ovx had significantly lower relative uterus weight (0.5±0.1 mg/g body weight) than E_2_-treated group (5.0±0.7 mg/g body weight, p<0.001), this confirming that ovariectomy and E_2_ replacement were successful. As shown in Figure 4A, tumor histology was consistent with that of poorly differentiated HGSOC. Noteworthy E_2_-treatment increased HEY tumor growth rate *in vivo*, resulting in a significant survival disadvantage compared to Ovx control mice (P<0.05). Overall, the pattern of ERs expression was similar to that observed in the NIH:OVCAR-3 study, with the exception of an increase in nuclear ERβ2 levels shown by E_2-_treated mice compared to Ovx control. (P<0.05) (Figure 4B). GPER expression did not differ between study groups (data not shown). Consistently with the NIH:OVCAR-3 model, proliferation rate was increased in Ovx+E_2_ females, although this difference did not achieve statistical significance (Figure 4C).

### Mechanistic studies

**Figure 5:**
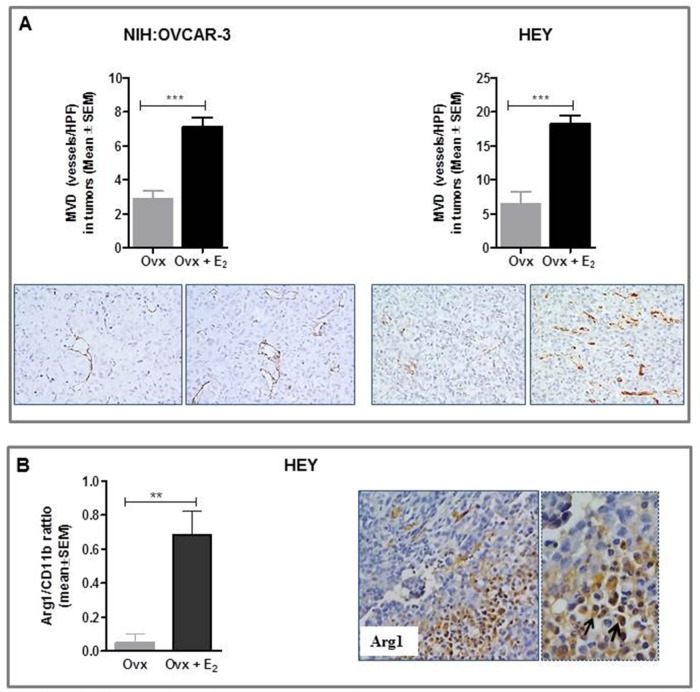
Effect of E_2_ on tumor angiogenesis and intratumoral TAM densities in in vivo preclinical models of ovarian cancer **A.** Immunohistochemical analysis showed in both experimental models a significant higher MVD (Microvessel Density) in E_2_-treated (***P<0.001, n=8 tumors/group) compared to Ovx females. Representative images for CD31 immunostaining of tumors from Ovx and Ovx+E_2_ mice (magnification 40x). **B.** Representative pictures for immunohistochemical staining of Arginase I-positive macrophages (Arg1) in HEY xenograft from E_2_-treated mice. Magnification 20x and 40x. Results obtained showed a significant increase of the ratio Arg1/CD11b in E_2_-treated compared to untreated Ovx mice (**P<0.01, n=8 tumors/group).

Overall, *in vivo* studies showed that increasing the levels of circulating estrogens resulted in an accelerated growth of ERα-negative HGSOC cells in nude mice. The lack of consistency between *in vitro* and *in vivo* data prompted us to investigate whether estrogens, besides a direct role in tumor proliferation, could promote the development and progression of ERα-negative tumors by changing the composition and nature of the tumor microenvironment. To this end, we assessed microvessel density in xenografts using antibody against CD31, a specific and sensitive endothelial marker for formalin-fixed paraffin-embedded tissues [32]. Notably, results demonstrated that estrogens indeed induced tumor angiogenesis in both experimental models, promoting a dense network of vessels with multiple branching (Figure 5A). Specifically, in NIH:OVCAR-3 xenografts, intratumoral MVD values of 7.2 ± 0.5 *vs* 2.9 ± 0.4 were detected in E_2_-treated and Ovx females, respectively (P< 0.001). Correspondingly, in HEY tumors values of 18.3 ± 1.1 and 6.5 ± 1.7 vessels/HPF were found in E_2_- treated and Ovx groups, respectively (P<0.001).

An extensive body of research has implicated estrogens in promoting angiogenesis, but few recent studies provide proof-of-concept data for a role of E_2_ in cancer pathogenesis by increasing the mobilization and recruitment of a proangiogenic population of bone marrow-derived cells (BMDCs). This results in enhanced macrophages that home to the tumor and initiate vasculogenesis and angiogenesis [33]. Macrophages constitute an extremely heterogeneous population which differentiate into distinct types, schematically identified as M1 (or classically activated) and M2 (or alternatively activated) [34]. “Classically activated” M1 macrophages contribute to tumor rejection through type 1 cytokine production and antigen presentation, whereas “alternatively activated” M2 macrophages enhance angiogenesis and remodeling, through type 2 cytokine production. It is now generally accepted that tumor-associated macrophages (TAM) most closely resemble M2-polarized cells, creating an immunosuppressive microenvironment and finally promoting tumor invasion, angiogenesis, and metastasis [34]. On the basis of these findings we used two murine monocyte/macrophages markers, CD11b (total macrophage density), and Arginase I (M2-polarized macrophages) [35–37] to clarify the possible relationship between estrogen exposure and M2 polarization in mice-derived xenografts. Notably, in the HEY model, we observed a significant increase in intratumoral TAM density expressed as Arg1/CD11b ratio in estrogen-treated females compared to untreated Ovx (P<0.01, Figure 5B). TAM accumulation was mostly observed in hypoxic/necrotic areas in tumors (Figure 5B). Similar data were obtained in the NIH:OVCAR-3 model (data not shown). Overall, our results suggest a role for endogenous estrogens in mediating recruitment and/or activation of macrophages at the tumor site.

### Human studies

To add translational value to our preclinical studies suggesting a role for estrogens in inducing monocyte/macrophage recruitment and polarization in HGSOC, we assessed expression of CD68 and CD163 in surgically collected human HGSOC specimens. Indeed, CD68 and CD163 are both used to identify macrophages in tissue section, but while CD68 is commonly used as a pan-macrophage marker, CD163 is regarded as a highly specific marker for M2-polarized macrophages in several human tumors, including ovarian cancer [37–44]. To the aim of the study, cases were stratified according to menopausal status (pre- *vs* post-menopausal), and to the absence or presence of the ERα protein in the epithelial tumor compartment (ERα-negative *vs* ERα-positive). Immunohistochemical data on ERα status of these tumors were available from a previous study [28]. Tumors were dichotomized as ERα negative for a score below or equal to 2, and as positive for a score higher than 2 (see Materials and Methods section for scoring system), as previously reported [28, 45]. Eighteen patients were premenopausal (median age 45, range 33-54) and 30 postmenopausal (median age 65, range 51-81). There were 8 ERα-negative and 10 ERα-positive tumors in premenopausal, and 5 ERα-negative and 25 ERα-positive tumors in postmenopausal cases, respectively.

**Figure 6:**
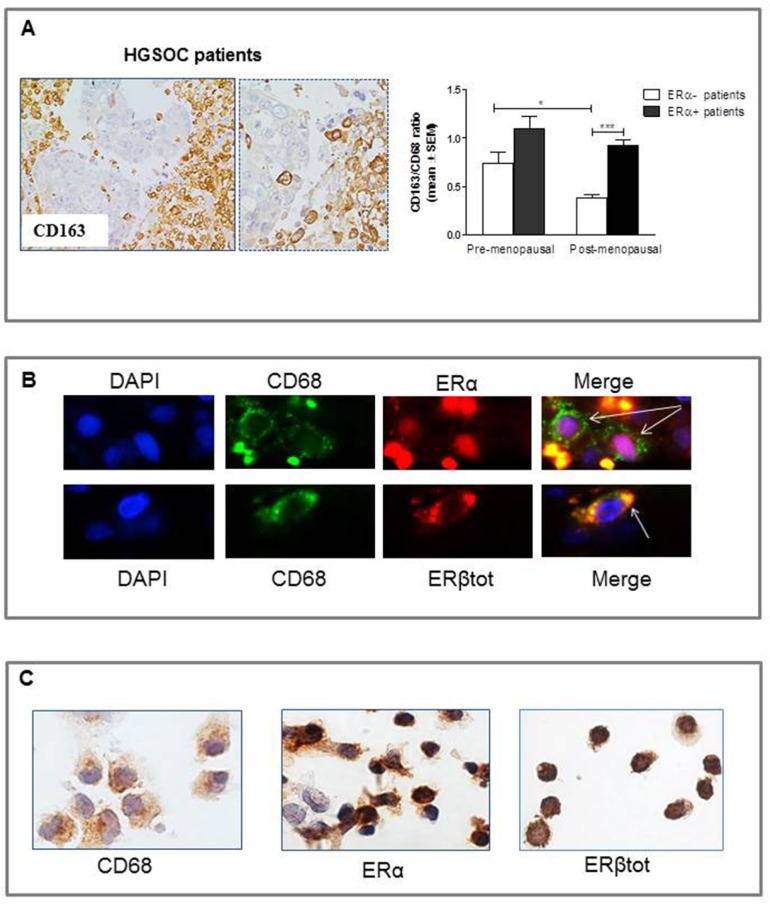
Densities of tumor associated macrophages (TAM) in 48 HGSOC tissue specimens **A.** Representative pictures for immunohistochemical staining of CD163+ macrophages in clinical samples of HGSOC. Magnification 20x and 40x. When considering only ERα-negative tumors, premenopausal patients showed a significant higher CD163/CD68 ratio compared to postmenopausal ones (*P<0.05; n=8 for pre-and n=5 for post-menopausal women). Notably, the highest levels of TAM infiltration were found in ERα-positive tumors irrespective of menopausal status (n=10 for pre-and n=25 for post-menopausal women). ***P<0.001. **B.** Pictures showing immunolocalization of ERα and ERβ in intratumoral macrophages (indicated by arrows) in HGSOG specimens. Sections were immunostained with the combination of anti-CD68 and anti-ERα/ERβtot antibodies. Nuclei were stained with DAPI (blue). Intratumoral macrophages were identified by green fluorescence of the monocyte/macrophage marker CD68. Superimposed images demonstrated a nuclear localization of ERα (red fluorescence) (pink coloration in Merge panel), while ERβ (red fluorescence) displayed mainly cytoplasmic expression in macrophages (yellow coloration in Merge panel). **C.** Representative pictures for immunostaining of monocytes isolated from human peripheral blood. Circulating monocytes, as confirmed by CD68 expression, showed a positive staining for ERα and ERβ.

As shown in Figure 6A, and in line with our preclinical findings and previous literature data, CD163-positive cells were mainly located in hypoxic/necrotic areas in tumors, a feature that has itself been linked to tumor aggressiveness [46]. Results showed that in the subgroup of ERα-negative tumors, a significantly higher TAM infiltration (expressed as CD163/CD68 ratio) was found in premenopausal compared to postmenopausal patients (P<0.05, Figure 6A), this implying that systemic effects of estrogens impact the tumor microenvironment, increasing the population of locally activated macrophages. Notably, however, data from clinical samples also showed that ERα-positive tumors had the highest levels of intratumoral TAM density, irrespective of menopausal status, statistical analysis showing that the difference in the CD163/CD68 ratio between ERα-negative and ERα-positive specimens achieved significance in postmenopausal patients only (P<0.01, Figure 6A).

We then used immunofluorescence to evaluate the expression of ERα and ERβ in human TAMs. As shown in Figure 6B, analysis indeed demonstrated that ERα or ERβ positive macrophages can be detected in clinical samples of HGSOC. However, the limited number of ERs+ macrophages observed in tumor specimens prompted us to investigate whether human circulating monocytes constitute a key target of systemic estrogens that, via their receptors, can recruit bone marrow-derived cells to the tumor site. To this end, monocytes were isolated from human peripheral blood and subsequently stained for ERα and ERβ expression, results showing that the majority of circulating CD68+ monocytes indeed expressed both ERs at similar levels (Figure 6C).

## DISCUSSION

Here we report novel findings on the role of estrogen signaling in the pathogenesis of HGSOC, demonstrating that E_2_ may stimulate tumor growth throughout multiple direct and indirect mechanisms. Indeed, although the role of estrogens in the progression of ovarian cancer has been classically related only to their mitogenic effects on ERα-expressing tumor cells [8, 47], we here demonstrated that additional, tumor-promoting activity could involve host tissue, thus ultimately inducing the growth acceleration of ERα-negative ovarian cancer, as well.

In line with the recognized proliferative role of ERα in ovarian cancer [8], our *in vitro* results actually confirmed that ERα expression is necessary and sufficient to induce the growth of HGSOC cells following estrogen treatment, since only the ERα-positive PEO1 cell line was growth-stimulated by E_2_ and PPT. On the other hand, no changes occurred in the ERα-negative NIH:OVCAR-3, COV318 and HEY cell lines after dosing with E_2_ or selective synthetic ligands, this showing that the activation of ERβ- or GPER-mediated signaling pathways does not elicit any significant changes in HGSOC cell growth.

Unexpectedly, *in vivo* studies demonstrated that increasing the levels of circulating estrogens resulted in an accelerated growth of ERα-negative ovarian cancer, as well. The analysis of estrogen receptor profile in xenografts a) confirmed the lack of ERα expression in the epithelial tumor component, thus indicating that tumor formation *in vivo* was not the results of acquired ERα expression, b) showed no differences between experimental groups in either ERβ1, ERβ5 and GPER protein level, and c) revealed an increased nuclear ERβ2 expression in E_2_-treated females (in the HEY model only). This latter finding, along with our preliminary data on A2780 tumors [12], supports the hypothesis that estrogens may actually affect both levels and subcellular distribution of ERβ2, that in turn appear to be associated with cell survival and/or estrogen-response. In line with this theory, emerging evidence suggests an oncogenic role for ERβ2, in contrast to the tumor suppressive effects of ERβ1 [13, 48, 49]. Despite of this, the discordance between *in vitro* and *in vivo* E_2_ effects on cell proliferation essentially indicates that circulating estrogens promote the outgrowth of ERα-negative cancers by influencing host cell types distinct from the epithelial ovarian cancer cells. Since the induction of angiogenesis is a crucial phase in the growth and progression of most solid tumors we were then prompted to assess the microvessel density (MVD) in xenografts using CD31, results indeed showing a significantly higher intratumoral MVD in E_2_-treated compared to Ovx females. Notably, in the last years there have been suggestions about the association between macrophages and tumors, with studies providing proof that tumor-associated macrophages can indeed promote angiogenesis (and tumor growth) through multiple mechanisms [50–52]. We therefore studied the density and distribution of macrophages in mice-derived xenografts, showing that a rise in estrogen circulating levels was associated with increased alternatively activated macrophages (M2-like) density, and their accumulation in hypoxic/necrotic areas in tumors. Notably, previous studies have demonstrated that that murine monocytes/macrophages express both ERs, with ERα being greater than ERβ [33, 53]. To the best of our knowledge, our data firstly demonstrate a central role of estrogens on the growth and dissemination of ERα-negative ovarian cancer, by increasing the population of locally activated macrophages at the tumor site, thus ultimately fostering a microenvironment that drives tumor progression. Our results are in line with recent data on breast cancer, providing evidences for a role of estrogens in promoting ER-negative tumor formation by influencing the mobilization and recruitment of a pro-angiogenic population of bone marrow-derived cells; a functional ERα in the BMDCs has been deemed necessary for this effect [33, 54].

To validate our preclinical results in a clinical setting, we assessed local macrophage polarization state in a series of HGSOC specimens stratified according to menopausal status and ERα expression in the epithelial compartment of tumor. Noteworthy, in keeping with preclinical data, in the subgroup of ERα-negative tumors, a significantly higher TAM infiltration was found in premenopausal compared to postmenopausal patients, this confirming a role of systemic estrogens in altering tumor microenvironment, independently of their direct effect on tumor cell growth. Moreover, recent work has begun to characterize expression and role of ERα and ERβ proteins in human monocytes and macrophages demonstrating that ERα is predominantly expressed in human macrophages [55], and that this receptor is mainly involved in macrophage polarization from a range of sources to an alternative phenotype [53]. Our immunofluorescent analysis on clinical specimens of HGSOCs confirmed the presence of both ERs in TAMs, although only in a limited number, the majority of them being negative for both proteins. On the other hand, we found a relevant expression of ERα and ERβ in circulating monocytes, which indeed could represent a target population for circulating estrogens, as suggested by previous studies [33, 54]. Besides, a role of systemic estrogens in controlling the macrophage-polarizing signaling has been recently reported by Toniolo [56] showing that M2-associated stimuli and activation is markedly impaired in macrophages from postmenopausal compared to premenopausal women.

Data from clinical samples also showed that ERα-positive tumors had the highest levels of intratumoral TAM density, irrespective of menopausal status, thus adding another layer of complexity to an already complicated picture. While only slight changes were observed between ERα-negative and ERα-positive tumors in premenopausal patients (possibly due to a prevailing effect of circulating estrogens on monocyte recruitment), in postmenopausal patients the difference in TAM infiltration between the two sub-populations reached statistical significance. These results suggest that in ERα-positive ovarian cancer, local estrogens may affect the communication between cancer cells and the surrounding stroma, further promoting TAM accumulation in the tumor microenvironment. Our hypothesis is supported by evidence on local generation of estrogens in ovarian cancer [5, 57], as well as by experimental data in breast cancer models showing that estrogens affect the communication between cancer cells, macrophages, and tumor blood vessels leading to increased cancer growth and metastasis [58]. Specifically, Svensson and colleagues demonstrated that E_2_ and the selective ERα agonist PPT were able to increase the release of the monocyte-attracting chemokines CCL2 and CCL5 by the ERα-positive MCF-7 cells (an effect not induced by treatment with DPN, a selective ERβ agonist), while chemokine levels were unaffected when the ERβ-positive MDA-MD-231 cells were treated with E_2_ or DPN. In *in vivo* models the increase in CCL2 and CCL5 was accompanied by a massive influx of macrophages and by a protumorigenic activation of the macrophages [58].

Overall, our findings suggest that the cellular and molecular mechanism for the generation of TAMs may be more complicated than we expected and, certainly, further research is needed to unravel the role of estrogens in this process. In this context, in the last decade there have been several reports showing that TAMs represent an important component of the ovarian tumor microenvironment and the most abundant infiltrating immune population in human ovarian tumors and ascites [59]. Besides, mechanistic investigations have shown that TAMs can promote the invasiveness of ovarian tumor cells through multiple mechanisms, and, in turn, ovarian cancer cells produce a variety of factors that induce recruitment and differentiation of macrophages with tumor-promoting functions [59]. In line with these results, expression of specific M2-associated markers in ovarian cancer indicates that TAMs can also predict patient prognosis in specific histopathological subtypes [59].

In conclusion, our data support the hypothesis that multiple direct and indirect mechanisms drive estrogen-induced tumor growth in HGSOC, showing that estrogen effect is not limited to ER-positive disease, but may largely proceed indirectly through the recruitment and activation of macrophages that have tumor-promoting functions at the tumor site. Defining the underlying mechanisms associated to estrogen exposure may have important implications for disease risk assessment, diagnosis, and treatment by refining antiestrogen-target drugs.

## MATERIALS AND METHODS

### Cell culture

The ovarian carcinoma cell lines COV318 and PEO1 were purchased from the European Collection of Cell Cultures (ECACC, Salisbury, UK); NIH:OVCAR-3 was purchased from the CLS Cell Lines Service GmbH (Eppelheim, Germany); and HEY cells were donated by Susan Horwitz (Albert Einstein Medical College, USA). PEO1, HEY and NIH:OVCAR-3 were grown in RPMI 1640 medium (Lonza, Basel, Switzerland), while COV318 were cultured in Dulbecco's modified Eagle's medium (Lonza). The medium was supplemented with 10% fetal bovine serum (FBS, Lonza), 2 mM glutamine and antibiotics (100 mg/ml streptomycin and 100 IU/ml penicillin) (Lonza). Insulin 0.01 mg/mL was also added to NIH:OVCAR-3 medium. All cultures were maintained at 37°C under a humidified atmosphere of 5% CO2 and 95% air.

### Western blot analysis

Whole-cell extracts were prepared after lysis in buffer containing 50 mM Tris-HCl, pH 7.4, 0.4 M NaCl, 1 mM EDTA, 1 mM EGTA, 1% Triton X-100, 0.5% NP-40, 10% Glycerol, supplemented with phosphate and protease inhibitors. Equal amounts of protein (60 μg/sample) were separated by SDS polyacrylamide gel electrophoresis (4-20%) (Bio-Rad Laboratories, Hercules, CA, USA) and transferred onto PVDF membranes (Immobilon-P transfer membrane, Millipore, Billerica, MA, USA). The membranes were blocked for 1 hour with 5% (w/v) nonfat dry milk (Bio-Rad Laboratories) in Tris Buffered Saline containing 0.1% Tween 20 (TBST), and then incubated with primary antibodies: anti-ERα (clone 6F11, Santa Cruz, CA, USA); anti-ERβ total (clone H150, Santa Cruz); anti-PR (clone 16, Leica Microsystems, Bannockburn, IL, USA); anti-GPER (clone N-15, Santa Cruz); anti-AR (clone AR441, Abcam, Cambridge, UK); anti-cyclin D1 (clone 92G2, Cell Signaling Technology, Leiden, The Netherlands); anti-Cyclin E (clone M20, Santa Cruz); anti-β-actin (A5441, Sigma-Aldrich, St. Louis, MO, USA) at 4°C overnight. After washing three times with TBST, the membranes were labeled with horseradish peroxidase-conjugated secondary antibodies for 1 hour at room temperature. Specific proteins were detected by the ECL Western blotting system according to the manufacturer's instructions (Pierce Biotechnology, Rockford, IL, USA). Immunoreactive bands were visualized using a VersaDoc (Bio-Rad Laboratories) imaging system, and quantitation was carried out using the Quantity One software program. β-actin was used as loading control in western blot analysis.

### Real time PCR

Total RNA was extracted using Total RNA isolation NucleoSpin RNA II (Macherey-Nagel GmbH & Co. KG, Germany) and was reverse-transcribed using RETROscript kit (Ambion®, Life Technologies Invitrogen, Carlsbad, CA, USA), according to the manufacturer's protocol. To evaluate hormone-receptor mRNA levels, each RT reaction mixture was subjected to real time PCR (qPCR) using SYBR® Green Master Mix (Bio-Rad Laboratories) in the following cycling conditions: 40 cycles of denaturation at 95°C for 15 s; annealing and extension at 60°C for 45 s. PCR reactions were carried out in a 25 μL reaction volume in a Bio-Rad real time PCR machine (CFX Connect). The PCR primers for detecting specific transcripts are reported in Table 1. All samples were amplified in triplicate and normalized to the housekeeping gene, GAPDH. Relative mRNA concentrations were calculated from the take-off point of reactions (threshold cycle, Ct) using the comparative quantitation method performed by Bio-Rad software, and based upon the -ΔΔCt method. In each assay, the PCR efficiency was also calculated using serial dilution of one experimental sample; efficiency values between 80 and 100% were found for each primer set and taken into account for the comparative quantitation analysis.

**Table 1:** Oligonucleotide primer sequences for real-time PCR

Gene	Forward Primers (5′→3′)	Reverse Primers (5′→3′)	Expected size (bp)
**ERα**	CCACCAACCAGTGCACCATT	GGTCTTTTCGTATCCCACCTTTC	108
**ERβ1**	GTCAGGCATGCGAGTAACAA	GGGAGCCCTCTTTGCTTTTA	181
**ERβ2**	AGGCATGCGAGGGCAGAA	GGCCACCGAGTTGATTAGAGG	115
**ERβ5**	GATGCTTTGGTTTGGGTGAT	CCTCCGTGGAGCACATAATC	165
**GPER**	AAACTGCGGTCAGATGTGGCT	TGTGTGAGGAGTGCAAG	117
**PR**	TCAGTGGGCAGATGCTGTATTT	GCCACATGGTAAGGCATAATGA	96
**AR**	AAGACGCTTCTACCAGCTCACCAA	TCCCAGAAAGGATCTTGGGCACTT	170
**GAPDH**	TCCCTGAGCTGAACGGGAAG	GGAGGAGTGGGTGTCGCTGT	218

### Proliferation assay

COV318 (2.8 × 10^5^ per well), NIH:OVCAR-3 (2.5 × 10^5^ per well), HEY (2.5 × 10^5^ per well) and PEO1 (5.0 × 10^5^ per well) cells were seeded in 6-well plates in complete culture medium. After overnight incubation, the medium was changed to phenol-free medium supplemented with 2% CS-FBS (charcoal-stripped serum, Life Technologies Invitrogen) and containing various concentrations of 17β-estradiol (E_2_, Sigma-Aldrich); 4,4′,4′′-(4-Propyl-[1H]-pyrazole-1,3,5-triyl) trisphenol (PPT, a selective ERα agonist, Tocris Bioscience, Ellisville, MO, USA); 2,3-bis(4-hydroxyphenyl)-propionitrile (DPN, a selective ERβ agonist, Tocris Bioscience); or (±)-1-[(3aR*,4S*,9bS*)-4-(6-Bromo-1,3-benzodioxol-5-yl)-3a,4,5,9b-tetrahydro-3H-cyclopenta[c]quinolin-8-yl]-ethanone (G1, a selective GPER agonist, Tocris Bioscience). The ER antagonist 7α,17β-[9-[(4,4,5,5,5-Pentafluoropentyl) sulfinyl]nonyl]estra-1,3,5(10)-triene-3,17-diol (ICI 182,780, Tocris Bioscience) was used in combination experiments when relevant. Substances were dissolved in absolute ethanol or DMSO and diluted in the appropriate culture medium immediately before use. Control cells received the same amount of diluent. The medium was renewed after 48 hours. At 120 hours of incubation, cells were harvested by trypsinization, and viable cells were counted using Nucleocounter (Chemometec, Allerod, Denmark). All experiments were performed at least three times in duplicate. To validate our experimental conditions, the proliferation of MCF-7 cells (ECACC) was assessed following treatment with E_2,_ PPT, DPN, and G1.

### Mice and tumor induction

Four-week-old female BALB/c nude mice were obtained from Charles River S.r.l. (Lecco, Italy), and housed under controlled conditions as previously described [60]. The UKCCCR guidelines for the welfare of animals in experimental neoplasia were followed [61]. Studies were approved by the Animal Care and Use Committee of the Università Cattolica del Sacro Cuore (Rome, Italy), and by the Italian Ministry of Health (Prot. CESA/A/3/2013). Since previous studies in our Department already demonstrated the tumorigenicity of both NIH:OVCAR-3 and HEY cells [62 and unpublished data], we firstly evaluated whether PEO1 and COV318 cells were suitable for development as murine xenografts. For characterization of tumor growth, up to 1 × 10^7^ cells were coinjected with Matrigel either intraperitoneally (i.p.) or subcutaneously (s.c.) into at least three female BALB/c nude mice and disease progression monitored for at least 90 days. In our hands, animals injected with either PEO1 or COV318 did not develop any visible i.p./s.c. disease after this period (data not shown). We thus assessed the role of estradiol in *in vivo* growth of NIH:OVCAR-3 and HEY cells.

For each experimental model, at six weeks of age, 16 female mice were anesthetized and bilaterally ovariectomized (Ovx); the success of ovariectomy was checked at necropsy by marked atrophy of the uterine horns. One week after ovariectomy, a group of Ovx mice (Ovx+E_2_; n=8) were implanted s.c. with 90-day release, 0.36 mg 17β-estradiol pellets (Innovative Research of America, Sarasota, FL, USA); these pellets are designed to produce 100-200 pg/ml of serum estradiol (as indicated by the supplier). The remaining Ovx females were left untreated (n=8). Xenografts were established by injecting 8 × 10^6^ cells in phenol red-free medium s.c. and i.p. for NIH:OVCAR-3 and HEY, respectively. Inoculated animals were observed daily. For the s.c. tumor model, growth was monitored by measuring tumors using vernier calipers and tumor weight was calculated from two dimensional measurements (mm): Tumor weight = length × width^2^/2 [60, 63]. Upon health decline (i.e., severe weight loss, paralysis, ruffling of fur, inactivity) mice were euthanized and autopsied. At necropsy, uteri were rapidly removed, freed of fat and weighted. All tumors were also removed and fixed in 4% buffered formalin for histology.

### Human studies

This retrospective study included 48 HGSOC specimens obtained from the Department of Pathology, School of Medicine, Catholic University of the Sacred Heart Rome, Italy. In our Institution a written informed consent is routinely requested from patients for collection of their clinical data, as well as paraffin embedded sections for research use. Clinical information was obtained from the existing medical records in accord with institutional guidelines. All data were managed using anonymous numerical codes.

### Immunohistochemistry

Immunohistochemical analysis was carried out on 3-μm thick paraffin sections as described [12]. Antibodies used include: anti-ERα (clone 6F11, Santa Cruz Biotechnology, dilution 1:100); anti-ERβ1 (clone PPG5/10, Dako, Glostrup, Denmark, dilution 1:50); anti-ERβ2 (clone 57/3, Serotec Ltd, dilution 1:200); anti-ERβ5 (clone 5/25, Serotec Ltd, dilution 1:300); anti-PR (clone 16SAN27, Leica Microsystems, dilution 1:100); anti-GPER (clone N-15, Santa Cruz, dilution 1:100); anti-Ki67 (clone MIB-1, Dako, dilution 1:100); anti-CD31 (ab28364, Abcam, dilution 1:50), anti-CD11b (Thermo Fisher scientific, Waltham, MA, USA, dilution 1:50); anti-arginase I (Santa Cruz Biotechnology, dilution 1:50); anti-CD68 (clone PG-M1, Dako, dilution 1:100) or anti CD163 (clone10D6, Biocare Medical, Concord, CA, USA, dilution 1:50).

### Evaluation of immunohistochemical staining

Scoring of ERs was evaluated as previously reported [12]. Briefly, the mean percentage of stained cells was categorized as follows: 0=negative, 1 = 1-10%, 2 = 11-33%, 3 = 34-66%, 4 = 67-100%. The intensity of staining was also evaluated and graded from 1-3, where 1=weak staining, 2=moderate staining, and 3=strong staining. The two values obtained were multiplied to calculate an immunoreactive score (IRS, maximum value 12). Positivity for Ki67 was evaluated by considering the number of cells exhibiting immunoreaction in a minimum of 500 histologically identified neoplastic cells. For the quantitative analysis of microvessel density, CD31-positive intratumoral microvessels were counted blindly under a microscope field (x400 objective magnification, high-power field area = 0.24 mm^2^). A minimum of 3 tumor areas per section were evaluated. The microvascular density (MVD) was expressed as mean number of vessel per high-power field (MVD, vessels/HPF). Finally, tumor-associated macrophage (TAM) densities were assessed by counting the number of intratumoral macrophages with positive staining for the phenotype marker(s) in four representative 400x high-power fields (total tumor surface: 1 mm^2^). Macrophage density was expressed as cells/mm^2^. Immunohistochemical assessment was carried out by two investigators blinded to groups.

### Immunofluorescence of fixed paraffin-embedded ovarian tissue sections

Three-micrometer-thick paraffin sections were mounted on Superfrost coated slides, and dried overnight. The sections were deparaffinized in xylene, rehydrated in graded solutions of ethanol and rinsed for 5 minutes in distilled water. After antigen retrieval procedure performed by microwave oven heating, the sections were incubated with 20% normal goat serum for 30 min at room temperature and then incubated at 4°C overnight with primary antibody: anti-CD68 (clone PG-M1, Dako, dilution 1:100); anti-ERα (clone SP1, Ventana Medical Systems, Inc. Tucson, Arizona, USA, pre-diluted); and anti-ERβtot (clone H150, Santa Cruz, 1:50). The optimal dilution of primary antibody had been established before by immuno-enzymatic staining. After overnight incubation, slides were washed in TBS and incubated in the dark for 1 hour at RT with secondary antibody anti-mouse Alexa Fluor-488 conjugate and anti-rabbit Texas Red (Life Technologies, Inc). After washing in PBS (Dulbecco's Phosphate Buffer Saline, Lonza), tissues were stained with DAPI (1.5 μg/ml) and mounted in Vectashield Mounting Medium (Vector Laboratories, Burlingame, Ontario, Canada). Slides were observed under the fluorescence microscope (Leica) using a 100X oil immersion objective.

### Isolation of monocytes from peripheral blood

Anonymous buffy coats from peripheral blood (PB) donations were collected from healthy blood bank donors (females, 18–65 years, n=3). Buffy coats were diluted (1:3) with phosphate-buffered saline, PBS (Lonza), layered over Ficoll-Hypaque (Sigma-Aldrich) density gradient and centrifuged at 1,800 rpm for 20 minutes. Freshly isolated mononuclear cells of PB (PBMC) were counted and re-suspended in RPMI complete medium containing 10% FBS, 2 mM L-glutamine, 10 UI/ml penicillin-streptomycin. 5 × 10^6^ cells/ml were seeded into chamber slides (Nunc® Lab-Tek® II Chamber Slide, Nunc, Inc., Naperville, IL) and monocytes were separated from lymphocytes by adherence to the surface after an incubation of 24h at 37°C, 5% CO2. Non-adherent cells were removed and adherent cells (>80% CD68+ by IHC) were washed and used for immunostaining analysis.

### Immunocytochemistry

Monocytes were washed twice with PBS, fixed and permeabilzed with Cytofix/Cytoperm Kit (BD Bioscences, Palo Alto, CA, USA), according to manufacturer's instructions. The endogenous peroxidase was blocked with 3% H_2_O_2_ for 5 min. After washing twice with PBS, cells were incubated with a blocking solution containing 20% normal horse serum in PBS for 30 min at room temperature. Excess blocking solution was drained, and samples were incubated with primary antibodies: anti-CD68 (clone PG-M1, Dako, dilution 1:100); anti-ERα (clone SP1, Ventana Medical Systems, Inc., pre-diluted); and anti-ERβ (clone 14C8, Novus Biologicals, dilution 1:30) overnight at 4°C in a humidified chamber. The samples were then rinsed three times with PBS and incubated with secondary antibody, EnVision System-HRP (Dako), for 30 min at room temperature. The immunoreactivity was detected using the 3,3′-diaminobenzidine substrate (DAB substrate System, Dako). The slides were counterstained with Mayer's Haematoxylin, dehydrated in ethanol and xylene, and finally mounted.

### Statistical analysis

For the NIH:OVCAR-3 model, tumor growth data were analyzed by repeated-measures ANOVA, followed by the Bonferroni method as post-test. For the HEY model, the Kaplan–Meier survival analysis was used, followed by log rank test. The remaining data were analyzed for homogeneity of variance using an F test. If the variances were heterogeneous, log or reciprocal transformations were made in an attempt to stabilize the variances, followed by Student's t-test. If the variances remained heterogeneous, a non-parametric test such as the Mann–Whitney U test was used. Data are reported as mean ± SEM. P values are for two-sided tests; p values ≤ 0.05 were considered statistically significant. Analyses were performed using GraphPad Prism version 5.0 for Windows (GraphPad Software, San Diego, CA).
